# Dynamics of Emergency Cardiovascular Hospital Admissions and In-Hospital Mortality During the COVID-19 Pandemic: Time Series Analysis and Impact of Socioeconomic Factors

**DOI:** 10.3389/fcvm.2022.827212

**Published:** 2022-04-26

**Authors:** Claudia Álvarez-Martín, Aida Ribera, Josep Ramon Marsal, Albert Ariza-Solé, Santiago Pérez-Hoyos, Gerard Oristrell, Toni Soriano-Colomé, Rafael Romaguera, Jose Ignacio Pijoan, Rosa M. Lidón, Josepa Mauri, Ignacio Ferreira-González

**Affiliations:** ^1^Cardiovascular Research and Epidemiology Unit, Cardiology Department, University Hospital Vall d’Hebron and Vall d’Hebron Research Institute, Barcelona, Spain; ^2^Centro de Investigación Biomédica en Red de Epidemiología y Salud Pública (CIBERESP), Madrid, Spain; ^3^Recerca en Envelliment, Fragilitat i Transicions (REFiT) Barcelona Research Group, Parc Sanitari Pere Virgili and Vall d’Hebron Institute of Research, Barcelona, Spain; ^4^Cardiology Department, Hospital Universitari de Bellvitge, Barcelona, Spain; ^5^Bioheart, Grup de Malalties Cardiovasculars, Institut d’Investigació Biomèdica de Bellvitge (IDIBELL), L’Hospitalet de Llobregat, Barcelona, Spain; ^6^Centro de Investigación Biomédica en Red en Enfermedades Cardiovasculares (CIBERCV), Madrid, Spain; ^7^Statistics and Bioinformatics Unit, Vall d’Hebron Research Institute, Barcelona, Spain; ^8^Statistics Department, Faculty of Biology, University of Barcelona, Barcelona, Spain; ^9^Cardiology Department, University Hospital Vall d’Hebron, Universitat Autònoma de Barcelona, Barcelona, Spain; ^10^Clinical Epidemiology Unit, Hospital Universitario Cruces/BioCruces-Bizkaia Health Research Institute, Barakaldo, Spain; ^11^Cardiology Department, Hospital German Trias i Pujol, Barcelona, Spain; ^12^Director Plan for Cardiovascular Diseases, Pla Director de Malalties Cardiovasculars (PDMCV), Department of Health, Catalan Government, Barcelona, Spain

**Keywords:** COVID-19, acute coronary syndrome, myocardial infarction, heart failure, time-series

## Abstract

**Aims:**

This study aimed to evaluate the decline in urgent cardiovascular hospital admissions and in-hospital mortality during the COVID pandemic in two successive waves, and to evaluate differences by sex, age, and deprivation index subgroups.

**Methods and Results:**

We obtained acute cardiovascular hospital episodes during the years 2019–2020 from region-wide data on public healthcare usage for the population of Catalonia (North-East Spain). We fitted time models to estimate the incidence rate ratios (IRRs) of the acute coronary syndrome (ACS) and acute heart failure (HF) admissions during the first pandemic wave, the between-waves period, and the second wave compared with the corresponding pre-COVID-19 periods and to test for the interaction with sex, age, and area-based socioeconomic level. We evaluated the effect of COVID-19 period on in-hospital mortality. ACS (*n* = 8,636) and HF (*n* = 27,566) episodes were defined using primary diagnostic ICD-10 codes. ACS and HF admissions decreased during the first wave (IRR = 0.66, 95%CI: 0.58–0.76 and IRR = 0.61, 95% CI: 0.55–0.68, respectively) and during the second wave (IRR = 0.80, 95%CI: 0.72–0.88 and IRR = 0.76, 95%CI: 0.69–0.84, respectively); acute HF admissions also decreased in the period between waves (IRR: 0.81, 95%CI: 0.74–0.89). The impact was similar in all sex and socioeconomic subgroups and was higher in older patients with ACS. In-hospital mortality was higher than expected only during the first wave.

**Conclusion:**

During the first wave of the COVID-19 pandemic, there was a marked decline in urgent cardiovascular hospital admissions that were attenuated during the second wave. Both the decline and the attenuation of the effect have been similar in all subgroups regardless of age, sex, or socioeconomic status. In-hospital mortality for ACS and HF episodes increased during the first wave, but not during the second wave.

## Introduction

During the first pandemic wave (FW) of coronavirus disease 2019 (COVID-19) between March and April 2020, there was a marked decline in admissions for acute coronary syndrome (ACS) (1–11), heart failure (HF) (12–14), and other non-COVID health conditions (15–17).

While prioritization of healthcare services explains decreases in elective admissions, explanations for the decrease in the attention of emergent conditions remain conjectural (18). Possible reasons attributed to the steep decrease in hospital admissions for acute cardiovascular problems were patients’ hesitation to refer to the hospital, missed diagnosis, and competing risk with COVID-19 mortality in the elderly and institutionalized patients (19). The analysis of differing trends of emergent cardiovascular admissions in successive waves periods and in the between-waves periods (BWP) might be useful to unravel the underlying mechanisms and to anticipate future trends. A few reports of results during the second pandemic wave (SW, between April and September 2020) indicate a second decline of unknown magnitude (20) despite information campaigns targeted at the affected populations.

Moreover, the COVID-19 pandemic has been shown to increase geographic, gender, and socioeconomic inequalities in access to healthcare (21). Age, gender, and deprivation level might influence behaviors in such ways that would imply a greater impact on more disadvantaged groups. Gender and age differences in admissions for acute myocardial infarction (MI) have been analyzed previously with differing results (22); but to the best of our knowledge, it has not been assessed in longer series (beyond the FW) considering socioeconomic indices. The potential influence of gender, age, and socioeconomic differences on the dynamic of admissions for acute HF decompensations has not been assessed so far.

Therefore, we aimed to evaluate the variations in ACS and acute HF hospital admissions during the COVID-19 pandemic period, including the FW and the SW, in comparison with the hospital admissions of the corresponding pre-COVID-19 pandemic periods. In addition, we aimed to assess whether age, gender, and socioeconomic status might have influenced these variations. Finally, we analyzed the predictors of in-hospital death.

## Materials and Methods

### Population and Data Sources

We used region-wide data on public healthcare usage for the population of Catalonia, a North-East region in Spain with universal public healthcare coverage, with a reference population of more than 7 M and a hospital network of 11 reference tertiary hospitals performing percutaneous coronary intervention (PCI). Data were accessed through the Public Data Analysis for Health Research and Innovation Program (PADRIS). PADRIS allows access to information from different sources linked at the patient level with warranted accomplishment of ethical principles and the Spanish Law of data protection 3/2018.

Acute coronary syndrome and HF episodes from 31 December 2018 to 27 December 2020 were extracted from the Minimum Basic Data Set (MBD). Index admissions were identified with the International Classification of Diseases (ICD)-10 as the primary diagnostic (see diagnostic codes in Supplementary Table 1). We selected only emergency admissions.

Weekly COVID-19 cases were obtained from the Open Access Data Portal of the Catalan Government. Confirmed cases were defined as having a positive RT-PCR test or rapid antigen test for SARS-CoV-2.

For each episode, we extracted sex, age in 5 years’ intervals, all diagnostic and procedure ICD-10 codes, region of residence, primary care service areas (PCSA), comorbidity status (AMG), and PCSA-based socioeconomic status (PCSA index).

### Primary Care Service Areas Index

To strengthen territorial equity in the allocation of primary care resources, in 2015, the Catalan Health Department developed a socioeconomic deprivation indicator representative of the PCSA linked to adverse health outcomes. The indicator score ranges from 0 (less deprived) to 100 (more deprived) (23).

### Comorbidity

The comorbidity index [Adjusted Morbidity Groups (AMGs)] score is a morbidity measure that enables assigning a weight based on the preexisting comorbidities (24). AMG explanatory value has been checked by comparing it with morbidity measures such as the Charlson Index or the number of chronic diseases (24).

### Statistical Analysis

Based on the visual representation of the weekly number of COVID-19 diagnostics, we defined the following periods of analysis: pre-COVID (from 31 December 2018 to 23 February 2020), FW (from 24 February 2020 to 27 April 2020), BWP (from 28 April 2020 to 20 September 2020), and SW (from 21 September 2020 to 27 December 2020). We considered the beginning of the FW as the date when the first case in the region was reported in the press (24 February 2020), although the COVID registry started later.

Patients’ characteristics were compared between each COVID period and the corresponding pre-COVID period (same calendar period in 2019). Continuous variables were reported as mean (SD) or median (P25 to P75) and compared using Student’s *t*-tests or Kruskal-Wallis tests. Categorical variables were reported as *n* (percentage) and compared using χ^2^ tests.

Weekly admissions for ACS and HF were depicted from 31 December 2018 to 27 December 2020. Negative binomial regression was used to estimate the incidence rate ratios (IRRs) and their 95% confidence intervals for ACS and acute HF hospital admissions during the COVID-19 FW, during the BWP, and during the SW compared with the expected admissions, based on the pre-COVID-19 period. Using this approach, each IRR represents the ratio between the weekly number of admissions in each period and the weekly number of admissions in the reference period. First, we modeled the pre-COVID-19 time series to estimate the expected number of admissions in the COVID-19 periods by fitting sinusoidal functions to control seasonality and autocorrelation. Best-fitted models were selected on the basis of minimizing deviance of the model and then fitted to the whole series including COVID waves as indicators to calculate IRR. To show graphically the variations in ACS and HF hospital admissions attributable to the pandemic phases, we depicted the observed vs. the expected admissions estimated from the pre-COVID-19 model.

Finally, we fitted an additional set of models using fractional polynomial coefficients to parameterize in a flexible way the different descending and ascending slopes observed during the pandemic period. We used the parameters of the fitted models to estimate the instantaneous rate of change in the different periods and the absolute decrease in each wave.

Different effects of COVID periods [FW, BWP, and SW among sex, age (<80/≥80), and PCSA index quartiles (first quartile/fourth quartile)] were estimated by including two-way interaction terms in regression models.

To assess whether changes in hospital mortality during the COVID-19 period were due to the distortion in healthcare during the pandemic or to changes in the risk profile of patients, we performed logistic mixed-effects regression with in-hospital mortality as the response variable and the pandemic periods together as a covariable. We adjusted the model by patients’ characteristics [e.g., sex, age, comorbidity weight, type of ACS (in ACS model), PCSA index quartiles]. We used random slope models and non-structured covariance matrix for random effects to account for repeated episodes of individual patients and for the clustered structure of patients living in different PCSA (371 PCSA). All analyses were performed with R version 4.1.0.

## Results

**FIGURE 1:**
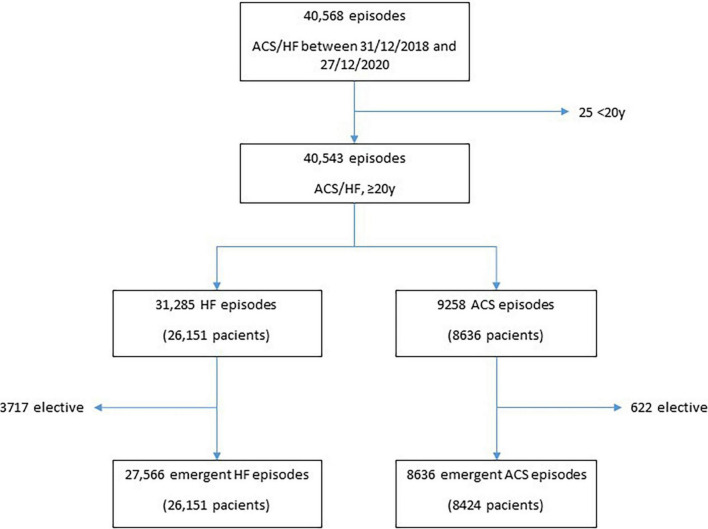
Flowchart.

After excluding elective admissions and patients younger than 20 years old, we analyzed a series of 36,202 episodes (corresponding to 34,575 patients) admitted for ACS (*n* = 8,424) or HF (26,151) between 31 December 2018 and 27 December 2020 (Figure 1).

Tables 1, 2 show the characteristics of patients attended for ACS and HF during each predefined COVID-19 period as compared with each corresponding pre-COVID-19 period: FW, BWP, and SW. The decline in admissions for every subgroup is presented as a% change. The description of the complete cohort is shown in Supplementary Table 2.

**TABLE 1:** Characteristics of patients admitted to hospital for acute coronary syndrome (ACS) in each period compared with the reference pre-COVID-19 period.

	First wave period (24/02 to 27/04)				Between waves period (28/04 to 20/09)				Second wave period (21/09 to 27/12)			
	Pre-COVID-19	COVID-19	% change	*P*-value	Pre-COVID-19	COVID-19	% change	*P*-value	Pre-COVID-19	COVID-19	% change	*P*-value
N	817	519	−36		1788	1308	−12		1308	1001	−23	
Women	250 (30.60)	171 (32.95)	−32	0.368	617 (34.51)	505 (32.21)	−18	0.159	449 (34.33)	331 (33.07)	−26	0.526
Men	567 (69.40)	348 (67.05)	−39		1171 (65.49)	1063 (67.79)	−9		859 (65.67)	670 (66.93)	−22	
Age				0.638				0.352				0.001
<80	579 (70.87)	374 (72.06)	−35		1288 (72.04)	1152 (73.47)	−11		883 (67.51)	738 (73.73)	−16	
≥80	238 (29.13)	145 (27.94)	−39		500 (27.96)	416 (26.53)	−17		425 (32.49)	263 (26.27)	−38	
Type of ACS				<0.001				<0.001				<0.001
Unstable angina	143 (17.50)	84 (16.18)	−41		272 (15.21)	274 (17.47)	1		167 (12.77)	167 (16.68)	0	
NSTEMI	560 (68.54)	325 (62.62)	−42		1249 (69.58)	997 (63.58)	−20		944 (72.17)	671 (67.03)	−29	
STEMI	97 (11.87)	57 (10.98)	−41		228 (12.75)	95 (6.06)	−58		173 (13.23)	63 (6.29)	−64	
Other MI	8 (0.98)	43 (8.29)	438		14 (0.78)	161 (10.27)	1050		10 (0.76)	75 (7.49)	650	
Other ACS	9 (1.10)	10 (1.93)	11		25 (1.4)	41 (2.61)	64		14 (1.07)	25 (2.50)	79	
AMG weight, mean (SD)	32.92 (17.46)	20.44 (16.29)	−38	<0.001	31.82 (16.47)	18.61 (14.44)	−41	<0.001	31.19 (16.27)	17.16 (13.85)	−45	<0.001
PCSA index, mean (SD)	42.11 (15.27)	42.68 (15.00)	1	0.514	42.20 (14.62)	41.24 (15.10)	−2	0.064	41.79 (14.56)	42.05 (14.56)	1	0.680
Quantiles of PCSA index				0.084				0.799				0.414
1st	202 (24.72)	116 (22.35)	−43		422 (23.60)	394 (25.13)	−7		324 (24.77)	221 (22.08)	−32	
2nd	188 (23.01)	113 (21.77)	−40		428 (23.94)	376 (23.98)	−12		306 (23.39)	262 (26.17)	−14	
3rd	187 (22.89)	139 (26.78)	−26		432 (24.16)	370 (23.60)	−14		305 (23.32)	236 (23.58)	−23	
4th	215 (26.32)	145 (27.94)	−33		472 (26.40)	389 (24.80)	−18		335 (25.61)	249 (24.88)	−26	
PCI during hospitalization	353 (43.21)	242 (46.63)	−31	0.220	826 (46.20)	633 (40.37)	−23	0.001	619 (47.32)	420 (41.96)	−32	0.010
In−hospital mortality	45 (5.51)	46 (8.86)	2	0.018	69 (3.86)	73 (4.66)	6	0.253	60 (4.59)	48 (4.80)	−20	0.814
Hospital length of stay (days), mean (SD); median (p25 to p75)	9.41 (8.03); 7 (5 to 11)	7.11 (6.34); 5 (3 to 9)	−24	<0.001	8.88 (7.71); 7 (4 to 11)	8.29 (7.21); 6 (4 to 10)	−7	<0.001	9.47 (9.63); 7 (4 to 11)	8.03 (6.97); 6 (4 to 10)	−15	<0.001

*Numbers indicate n (%) except if otherwise stated.*

**TABLE 2:** Characteristics of patients admitted to hospital for acute heart failure (HF) in each period compared with the reference pre-COVID-19 period.

	First wave period (24/02 to 27/04)				Between waves period (28/04 to 20/09)				Second wave period (21/09 to 27/112)			
	Pre-COVID-19	COVID-19	% change	*P*-value	Pre-COVID-19	COVID-19	% change	*P*-value	Pre-COVID-19	COVID-19	% change	*P*-value
N	3,200	1,749	−45		5,641	4,026	−29		4,090	2795	−32	
Women	1,741 (54.41)	946 (54.09)	−46	0.830	3130 (55.49)	2,200 (54.16)	−30	0.195	2,315 (56.60)	1,525 (54.56)	−34	0.094
Men	1,459 (45.59)	803 (45.91)	−45		2,511 (44.51)	1,862 (45.84)	−26		1,775 (43.40)	1,270 (45.44)	−28	
Age				0.107				0.001				0.061
<80	1,246 (38.94)	722 (41.28)	−42		2,106 (37.33)	1,651 (40.65)	−22		1,517 (37.09)	1,099 (39.32)	−28	
≥80	1,954 (61.06)	1,027 (58.72)	−47		3,535 (62.67)	2,411 (59.35)	−32		2,573 (62.91)	1,696 (60.68)	−34	
AMGweight, mean (SD)	48.33 (15.39)	33.86 (18.22)	−30	<0.001	48.74 (15.86)	31.18 (17.69)	−36	<0.001	46.40 (15.73)	28.08 (15.59)	−39	<0.001
PCSA index, mean (SD)	41.03 (15.08)	41.76 (14.66)	2	0.108	40.76 (15.27)	41.73(15.15)	2	0.002	40.73 (15.33)	40.96 (14.99)	1	0.556
Quantiles of PCSA index				0.166				0.110				0.751
1st	836 (26.13)	426 (24.36)	−49		1,534 (27.19)	1,008 (24.82)	−34		1,137 (27.80)	754 (26.98)	−34	
2nd	790 (24.69)	408 (23.33)	−48		1349 (23.91)	989 (24.35)	−27		995 (24.33)	681 (24.36)	−32	
3rd	681 (21.28)	419 (23.96)	−38		1,220 (21.63)	929 (22.87)	−24		861 (21.05)	573 (20.50)	−33	
4th	790 (24.69)	445 (25.44)	−44		1,370 (24.29)	1,006 (24.77)	−27		986 (24.11)	712 (25.47)	−28	
In−hospital mortality	237 (7.41)	162 (9.26)	−32	0.022	421 (7.46)	259 (6.38)	−38	0.039	312 (7.63)	166 (5.94)	−47	0.007
Hospital length of stay (days), mean (SD); median (p25 to p75)	10.33 (10.04); 8 (4 to 13)	8.64 (7.32); 7 (4 to 11)	−16	<0.001	9.59 (8.66); 7 (4 to 12)	8.98 (7.75); 7 (4 to 11)	−6	0.017	9.77 (9.34); 7 (4 to 12)	8.47 (7.13); 6 (3 to 11)	−13	<0.001

*Numbers indicate n (%) except if otherwise stated.*

In general, patients admitted during the pandemic period had less comorbidities, as reflected by a consistently lower AMG score compared with the pre-COVID periods. In addition, in both conditions, the total hospital length of stay was significantly lower. Regarding ACS admissions (Table 1), the decline was significantly higher for patients aged 80 years or older admitted during the SW. Diagnostic episodes labeled as other AMI or other ACS increased in the whole COVID-19 period. As compared with the pre-COVID-19 period, the number of PCI remained virtually the same during the FW but decreased slightly during the BWP (−23%; *p* = 0.001) and during the SW (−32%; *p* = 0.01). In-hospital mortality increased during the FW [8.86% vs. 5.51% in the pre-COVID-19 period (*p* = 0.018)], and there were no statistically significant differences neither during the SW nor in the BWP.

Regarding acute HF admissions (Table 2), the proportion of older people admitted during the BWP was significantly lower, and in-hospital mortality was higher during the FW (7.41 vs. 9.26%; *p* = 0.022) but lower during the BWP (7.46 vs. 6.38%; *p* = 0.039) and during the SW (7.63% vs. 5.94%; *p* = 0.007).

**FIGURE 2:**
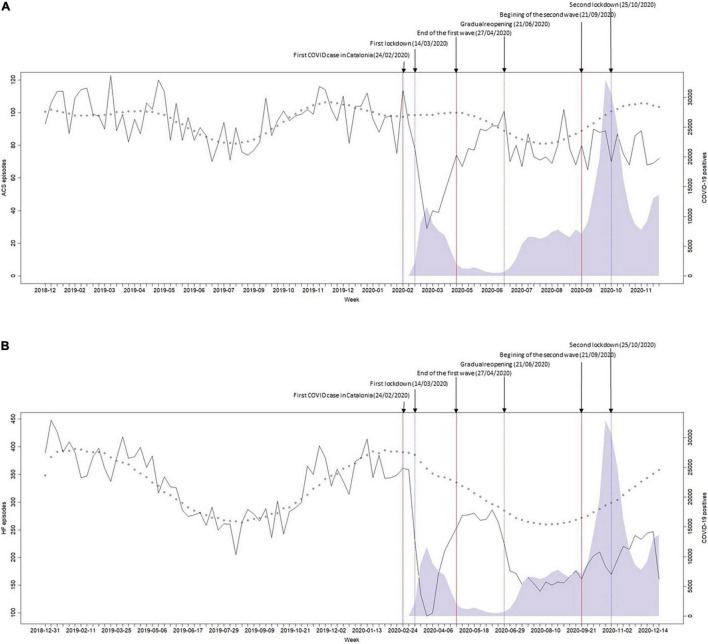
Weekly number of hospital admissions for **(A)** acute coronary syndrome and **(B)** heart failure, between 31 December 2018 and 27 December 2020. Black line: observed hospital admissions; shaded area: number of COVID-19 cases; red lines: predefined period limits: pre-pandemic, first wave, period between waves, and second wave; small circles: negative binomial model estimated values (pre-COVID-19).

Figure 2 shows the time series of ACS (Figure 2A) and HF (Figure 2B) weekly urgent hospital admissions and the distortion observed after the arrival of the pandemic wave. Both ACS and HF admissions series showed a significant seasonality, with lower admissions in summer weeks. After adjusting for seasonality and autocorrelation, there was a marked decrease in ACS episodes (Supplementary Figure 1A), reaching a maximum of 69.46% cases below the expected [IRR 0.43 (0.37–0.50), *p* < 0.001] coinciding with the highest peak of the FW. Then, after a recovery in the BWP, the number of admissions decreased again in a smoother way. Admissions for HF (Supplementary Figure 1B) also decreased steeply to a 72.83% less than expected [IRR 0.21 (0.17–0.26), *p* < 0.001] coinciding with the peak of the FW and then returned to normal levels to immediately decrease again to a sustained level about 30% below the expected.

The model parameters used to estimate IRRs and changes in the weekly number of admissions are shown in Supplementary Tables 3–6. The IRRs of ACS admissions for the FW, the BWP, and the SW were 0.66 (0.58–0.76), 0.92 (0.84–1.00), and 0.80 (0.72–0.88), respectively. The IRRs of HF admissions for the FW, the BWP, and the SW were 0.61 (0.55–0.68), 0.81 (0.74–0.89), and 0.76 (0.69–0.84), respectively. Supplementary Figures 2, 3 show the results of the models with fractional polynomic coefficients for ACS and HF.

**FIGURE 3:**
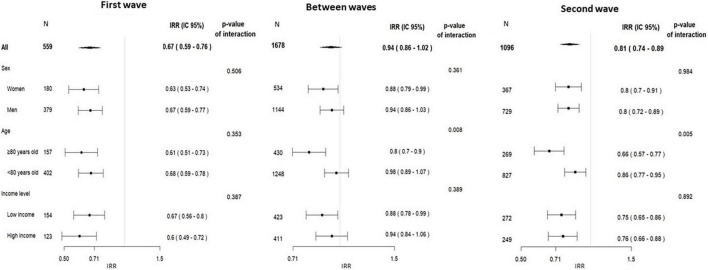
Incidence rate ratios of acute coronary syndrome admissions, in the three predefined pandemic period vs. the corresponding pre-pandemic period (2019) by subgroups of socioeconomic factors, and the *p*-value for the interaction between period and each socioeconomic factor.

**FIGURE 4:**
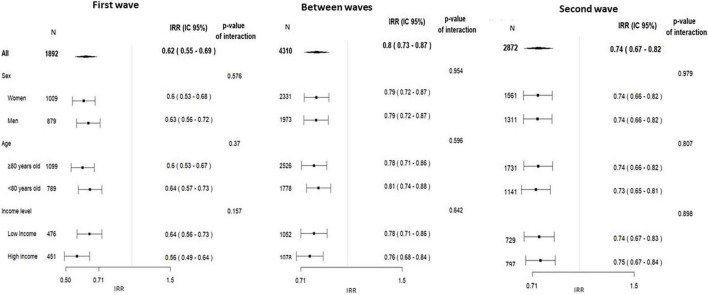
Incidence rate ratios of acute heart failure admissions, in the three predefined pandemic period vs. the corresponding pre-pandemic period (2019) by subgroups of socioeconomic factors, and the *p*-value for the interaction between period and each socioeconomic factor.

Supplementary Figures 4–15 show the separate time series according to gender, age, and area-level deprivation index. For the IRRs of ACS admissions, there was a significant interaction with age in the BWP and in the SW (Figure 3), when the relative reduction in the weekly number of admissions was higher for older people. Despite an apparent recovery in the admissions for ACS in the BWP [IRR 0.92 (0.84–1.00)], ACS admissions remained lower than expected among women, older, and in most deprived areas. Regarding HF, there was a significant decline of a similar magnitude in all subgroups (Figure 4), and HF admissions remained significantly lower than expected during the whole pandemic period.

Table 3 shows the association between the COVID-19 epidemic periods and in-hospital mortality adjusted for patient demographics, AMG score, type of ACS, and PCSA index. Mortality risk, compared with the corresponding pre-COVID-19 periods, was higher during the FW for both ACS (OR: 2.29; 95%CI: 1.6–3.27; *p* < 0.001) and HF episodes (OR: 1.33; 95% CI: 1.11–1.59; *p* = 0.002). In addition, ACS episodes labeled as other MI had the highest mortality risk (8.9; 95% CI 5.44–14.58; *p* < 0.001) as well as those episodes with a concomitant or recent diagnosis of coronavirus infection (OR: 4.1; 95% CI: 1.78–9.44; *p* = 0.001 and OR: 1.87; 95% CI: 1.22–2.86; *p* = 0.004 for ACS and HF, respectively).

**TABLE 3:** Association of patient characteristics and COVID-19 periods with in-hospital mortality for acute coronary syndrome (ACS) and heart failure (HF).

	ACS valid (n* = 8,622)		HF valid (n** = 27,531)	
	OR (95% CI)	*P*-value	OR (95% CI)	*P*-value
Women	0.93 (0.75 to 1.15)	0.487	0.86 (0.78 to 0.95)	0.002
Age (for 5 years increase)	1.44 (1.36 to 1.52)	<0.001	1.30 (1.26 to 1.34)	<0.001
AMG weight	1.02 (1.01 to 1.02)	<0.001	1.01 (1.00 to 1.01)	<0.001
Type of ACS				
Unstable angina (ref)	1		-	
Other ACS	2.81 (1.32 to 5.99)	0.007		
NSTEMI	2.22 (1.51 to 3.26)	<0.001	-	
STEMI	6.13 (4.03 to 9.35)	<0.001	-	
Other MI	8.67 (5.32 to 14.13)	<0.001	-	
Quartiles of PCSA index				
1st	1		1	
2nd	1.00 (0.75 to 1.33)	0.993	1.05 (0.90 to 1.23)	0.501
3rd	1.19 (0.90 to 1.57)	0.223	1.10 (0.94 to 1.28)	0.245
4th	1.09 (0.83 to 1.44)	0.525	1.05 (0.90 to 1.22)	0.553
COVID19 period				
1st wave	2.14 (1.50 to 3.05)	<0.001	1.36 (1.14 to 1.62)	0.001
Between waves	1.20 (0.90 to 1.62)	0.217	0.94 (0.82 to 1.09)	0.432
2nd wave	1.37 (0.97 to 1.93)	0.072	0.86 (0.72 to 1.03)	0.094

*14 missing values for PCSA; **35 missing values for PCSA.

*ACS, acute coronary syndrome; HF, heart failure; OR, odds ratio; AMG, adjusted morbidity groups; NSTEMI, non-ST elevation myocardial infarction; STEMI, ST elevation myocardial infarction; MI, myocardial infarction; PCSA, primary care service areas.*

## Discussion

This population-based time-trends analysis shows an important decrease in the number of emergent admissions for ACS and HF during the FW and during the SW compared with the corresponding pre-COVID-19 periods. This decrease was also present during the BWP, and it was similar among socioeconomic subgroups. However, there were less patients aged 80 years or older admitted for ACS during the BWP and the SW. Adjusted in-hospital mortality risk compared with the corresponding pre-COVID-19 periods was higher during the FW for both ACS and HF episodes despite a lower comorbidity weight and a similar rate of PCI.

Although the decrease in cardiovascular admissions was consistent during all the study periods, the impact of the SW was clearly different from the impact of the FW. The impact of the SW was quite attenuated, probably due to the rapid reaction of hospitals to increase their intensive care capacity and to the information campaigns launched to raise awareness of the need to contact the emergency services when experiencing symptoms compatible with MI.

There were also remarkable differences between ACS episodes and acute HF episodes. While the number of ACS admissions gradually returned to the expected levels in the BWP, admissions for acute HF remained low for almost the whole observed pandemic period studied. Whether the number of admissions has recovered to usual values or has decreased again to similar levels in the successive waves needs to be further addressed. Besides patients’ hesitation to go to the hospital, this sustained decrease might be related in part to the fact that HF symptoms are closer to COVID-19 symptoms. Since the primary focus of health professionals has been a rapid COVID-19 diagnosis, it might have led to underdiagnoses of HF cases. In addition, acute HF hospitalizations might have been lower because patients were preferably attended to in the primary care setting or due to the increased use of telemedicine, which is less practicable in the setting of the acute ischemic heart disease.

It has been speculated that lifestyle changes occurring during the lockdown periods might have influenced acute coronary disease incidence (25). However, the observations, as in other contexts (8), that the decline in admissions preceded the lockdown and that the return to “normal” levels preceded the reopening suggest that environmental changes, decreased physical activity, or diminished stress are unlikely to be major contributors to these trends.

Some changes in the characteristics of patients attended to during the successive COVID-19 periods compared with the corresponding pre-COVID-19 periods might give some hints for the interpretation of trends. Overall, patients admitted during the COVID-19 period had much lower comorbidity weight assessed by AMG score. This suggests that those patients with more comorbidities and higher risk who had a cardiovascular event did not seek care or that the healthcare system collapsed at some point and could not hospitalize these more fragile patients. Another possibility is a certain degree of competitive risk between COVID-19 disease and cardiovascular events, which could result in a lower rate of cardiovascular events in the population with a higher rate of severe COVID-19 disease, which was precisely the elderly and the more fragile population, especially during the FW. The stronger decrease in admissions of patients aged 80 years or older in the BWP and during the SW supports the hypothesis of competitive risk with the high COVID-19-related mortality during the FW.

Another indirect finding that suggests the burden of the healthcare facilities during certain periods was the hospital length of stay. It declined steadily during the epidemic waves and, to a lesser extent, during the BWP. In the future, it will become clear whether this indicates a simple trend in a complex context or a real change in the efficiency of the health system for managing these cardiovascular events. In any case, it indicates the effort of the healthcare professionals to minimize the overload of the system and the awareness of the risk of infection during admission by professionals and by the patients and their families.

The increase in the number of diagnoses labeled as other ACS or other MI, present in only few cases, might be due to codification problems during the overwhelming working conditions of the professionals responsible for codification during the pandemic peaks. This fact prevents us to draw conclusions about the differential changes by type of ACS.

The analysis of mortality determinants reveals that, unlike the results observed in other series (26), there was a significantly increased risk of in-hospital mortality after ACS and acute HF episodes in the FW but not in the SW. The increase in mortality was independent of case mix and despite a lower comorbidity weight and a similar rate of PCI compared with the pre-COVID-19 period. This probably reflects, on the one hand, the delay in patients seeking care increasing symptoms-to-balloon time (2), together with a more important healthcare overload during the FW and, on the other hand, a certain learning curve effect that benefited the second and subsequent waves.

Our results are consistent with those observed in other contexts. The study by Bodilsen et al. (16), in Denmark, highlighted HF as one of the health conditions not returning to baseline levels, together with respiratory and nervous system diseases, cancer, acute exacerbations of chronic pulmonary disease, sepsis, and pneumonia.

Although female sex and older age have been linked to increased delay before seeking healthcare (27), the COVID-19 pandemic does not seem to have affected differently the pattern of admission for ACS and HF in male and female patients (Figures 3, 4). This suggests that the potential different behavior of men and women for seeking care shown in other studies is attenuated in a context such as COVID-19 pandemic. However, we observed that the decline in hospital admissions was higher in older patients, but only after the FW. Huynh et al. (28) found a higher reduction of acute MI in women ≥70 years old during the COVID-19 pandemic in a Swedish healthcare region without lockdown.

To the best of our knowledge, this is the largest study showing the effects of the COVID-19 pandemic on acute cardiovascular disease admissions during a sufficiently large time window to include two successive waves. Previous reports have observed lower hospital admission rates for MI (5, 6, 11, 29, 30) and HF (13, 14, 31, 32) during the FW that are comparable with our study; however, reports of further declines in successive waves are scarce. In this sense, Wu et al. (20) reported a second decline of a similar magnitude (41% for HF and 34% for MI) during the SW in the United Kingdom.

### Limitations

Despite the use of large region-wide administrative databases that offer clear advantages, disadvantages include the following: Lack of detail in clinical severity, comorbidities, out-of-hospital mortality and causes of mortality, lower data quality, and higher risk of misclassification. In the case of HF, it would have been informative to merge hospital admissions to primary care emergent admissions and telecare attentions. Adoption of telecare programs as a response to the pandemic might have influenced the rate of hospital admissions. Finally, we used related ICD-10 diagnostic codes to label cases with concomitant COVID-19, as we did not have access to data for laboratory-proven SARS-Cov-2 infection. However, the proportion of patients with a diagnosis of both cardiovascular disease and COVID-19 infection was low, and its impact on overall outcomes might not be very relevant. Finally, the depiction of the COVID wave does not correspond with the real cases, especially during the FW, when fewer RT-PCR tests or rapid antigen tests for SARS-CoV-2 were performed, and specific diagnostic codes for COVID-19 were not available.

## Conclusion and Practical Implications

The decline in hospital admissions for ACS and acute HF during the FW of the COVID-19 pandemic was marked and striking. We provide evidence that the decline was attenuated but still observed in the SW and that the impact has been similar in all age, sex, and socioeconomic subgroups in the Catalan healthcare system. In addition, the adjusted in-hospital mortality risk comparing the corresponding pre-COVID-19 periods shows a clearly higher risk during the FW for both ACS and HF episodes but not during the SW, which indicates the difficulty of the healthcare system to adapt to new and stressful pandemic conditions.

The impact of COVID-19 in Spain was among the strongest reported worldwide, and its indirect impact on other non-COVID health conditions should be surveilled by health authorities. Numerous efforts have been made to forecast health resources needs for COVID-19 but not for other health problems that have been strongly impacted by the pandemic. Extending our temporal series to longer time frames, and including other contextual factors that might influence the rate of ACS or HF admissions, can serve to anticipate future distortions and prepare the health services to improve the attention of non-COVID health problems.

## Data Availability Statement

The data analyzed in this study is subject to the following licenses/restrictions: Restrictions apply to the availability of these data, which were used under license for this study. Data are available from the authors upon request with the permission of AQuAS. Requests to access these datasets should be directed to https://aquas.gencat.cat/ca/ambits/analitica-dades/padris/.

## Ethics Statement

The studies involving human participants were reviewed and approved by Clinical Research Ethics Committee Vall d’Hebron. Written informed consent for participation was not required for this study in accordance with the national legislation and the institutional requirements.

## Author Contributions

AR, AA-S, and IF-G conceived the study. AR received funds for the study. AR, JM, RL, and CÁ-M obtained the data. AR, CÁ-M, JRM, and SP-H performed the data management and statistical analysis. AR and IF-G have drafted the manuscript. All authors participated in the study design, contributed to the interpretation of the results, revised it critically for important intellectual content, and gave the final approval.

## Conflict of Interest

The authors declare that the research was conducted in the absence of any commercial or financial relationships that could be construed as a potential conflict of interest.

## Publisher’s Note

All claims expressed in this article are solely those of the authors and do not necessarily represent those of their affiliated organizations, or those of the publisher, the editors and the reviewers. Any product that may be evaluated in this article, or claim that may be made by its manufacturer, is not guaranteed or endorsed by the publisher.
