# Corrigendum: Dynamics of Emergency Cardiovascular Hospital Admissions and In-Hospital Mortality During the COVID-19 Pandemic: Time Series Analysis and Impact of Socioeconomic Factors

**DOI:** 10.3389/fcvm.2022.935850

**Published:** 2022-06-09

**Authors:** Claudia Álvarez-Martín, Aida Ribera, Josep Ramon Marsal, Albert Ariza-Solé, Santiago Pérez-Hoyos, Gerard Oristrell, Toni Soriano-Colomé, Rafael Romaguera, Jose Ignacio Pijoan, Rosa M. Lidón, Josepa Mauri, Ignacio Ferreira-González

**Affiliations:** ^1^Cardiovascular Research and Epidemiology Unit, Cardiology Department, University Hospital Vall d'Hebron and Vall d'Hebron Research Institute, Barcelona, Spain; ^2^Centro de Investigación Biomédica en Red de Epidemiología y Salud Pública (CIBERESP), Madrid, Spain; ^3^Recerca en Envelliment, Fragilitat i Transicions (REFiT) Barcelona Research Group, Parc Sanitari Pere Virgili and Vall d'Hebron Institute of Research, Barcelona, Spain; ^4^Cardiology Department, Hospital Universitari de Bellvitge, Barcelona, Spain; ^5^Bioheart, Grup de Malalties Cardiovasculars, Institut d'Investigació Biomèdica de Bellvitge (IDIBELL), L'Hospitalet de Llobregat, Barcelona, Spain; ^6^Centro de Investigación Biomédica en Red en Enfermedades Cardiovasculares (CIBERCV), Madrid, Spain; ^7^Statistics and Bioinformatics Unit, Vall d'Hebron Research Institute, Barcelona, Spain; ^8^Statistics Department, Faculty of Biology, University of Barcelona, Barcelona, Spain; ^9^Cardiology Department, University Hospital Vall d'Hebron, Universitat Autònoma de Barcelona, Barcelona, Spain; ^10^Clinical Epidemiology Unit, Hospital Universitario Cruces/BioCruces-Bizkaia Health Research Institute, Barakaldo, Spain; ^11^Cardiology Department, Hospital German Trias i Pujol, Barcelona, Spain; ^12^Director Plan for Cardiovascular Diseases, Pla Director de Malalties Cardiovasculars (PDMCV), Department of Health, Catalan Government, Barcelona, Spain

**Keywords:** COVID-19, acute coronary syndrome, myocardial infarction, heart failure, time-series

In the original article, there was a mistake in “Table 1” as published. The N for COVID-19 First wave period is 519 instead of 5,19. The corrected “Table 1” appears below.

**Table 1:** Characteristics of patients admitted to hospital for acute coronary syndrome (ACS) in each period compared with the reference pre-COVID-19 period.

	**First wave period (24/02 to 27/04)**				**Between waves period (28/04 to 20/09)**				**Second wave period (21/09 to 27/12)**			
	**Pre COVID-19**	**COVID-19**	**% change**	***P value***	**Pre COVID-19**	**COVID-19**	**% change**	***P value***	**Pre COVID-19**	**COVID-19**	**% change**	***P value***
N	817	519	−36		1788	1308	−12		1308	1001	−23	
Women	250 (30.60)	171 (32.95)	−32	0.368	617 (34.51)	505 (32.21)	−18	0.159	449 (34.33)	331 (33.07)	−26	0.526
Men	567 (69.40)	348 (67.05)	−39		1171 (65.49)	1063 (67.79)	−9		859 (65.67)	670 (66.93)	−22	
Age				0.638				0.352				0.001
<80	579 (70.87)	374 (72.06)	−35		1288 (72.04)	1152 (73.47)	−11		883 (67.51)	738 (73.73)	−16	
≥80	238 (29.13)	145 (27.94)	−39		500 (27.96)	416 (26.53)	−17		425 (32.49)	263 (26.27)	−38	
Type of ACS				<0.001				<0.001				<0.001
Unstable angina	143 (17.50)	84 (16.18)	−41		272 (15.21)	274 (17.47)	1		167 (12.77)	167 (16.68)	0	
NSTEMI	560 (68.54)	325 (62.62)	−42		1249 (69.85)	997 (63.58)	−20		944 (72.17)	671 (67.03)	−29	
STEMI	97 (11.87)	57 (10.98)	−41		228 (12.75)	95 (6.06)	−58		173 (13.23)	63 (6.29)	−64	
Other MI	8 (0.98)	43 (8.29)	438		14 (0.78)	161 (10.27)	1050		10 (0.76)	75 (7.49)	650	
Other ACS	9 (1.10)	10 (1.93)	11		25 (1.4)	41 (2.61)	64		14 (1.07)	25 (2.50)	79	
AMG weight, mean (SD)	32.92 (17.46)	20.44 (16.29)	−38	<0.001	31.82 (16.47)	18.61 (14.44)	−41	<0.001	31.19 (16.27)	17.16 (13.85)	−45	<0.001
PCSA index, mean (SD)	42.11 (15.27)	42.68 (15.00)	1	0.514	42.20 (14.62)	41.24 (15.10)	−2	0.064	41.79 (14.56)	42.05 (14.56)	1	0.680
Quantiles of PCSA index				0.084				0.799				0.414
1st	202 (24.72)	116 (22.35)	−43		422 (23.60)	394 (25.13)	−7		324 (24.77)	221 (22.08)	−32	
2nd	188 (23.01)	113 (21.77)	−40		428 (23.94)	376 (23.98)	−12		306 (23.39)	262 (26.17)	−14	
3rd	187 (22.89)	139 (26.78)	−26		432 (24.16)	370 (23.60)	−14		305 (23.32)	236 (23.58)	−23	
4th	215 (26.32)	145 (27.94)	−33		472 (26.40)	389 (24.80)	−18		335 (25.61)	249 (24.88)	−26	
PCI during hospitalization	353 (43.21)	242 (46.63)	−31	0.220	826 (46.20)	633 (40.37)	−23	0.001	619 (47.32)	420 (41.96)	−32	0.010
Inhospital mortality	45 (5.51)	46 (8.86)	2	0.018	69 (3.86)	73 (4.66)	6	0.253	60 (4.59)	48 (4.80)	−20	0.814
Hospital length of stay (days), mean (SD); median (p25 to p75)	9.41 (8.03); 7 (5 to 11)	7.11 (6.34); 5 (3 to 9)	−24	<0.001	8.88 (7.71); 7 (4 to 11)	8.29 (7.21); 6 (4 to 10)	−7	<0.001	9.47 (9.63); 7 (4 to 11)	8.03 (6.97); 6 (4 to 10)	−15	<0.001

*Numbers indicate n (%) except if otherwise stated*.

The authors apologize for this error and state that this does not change the scientific conclusions of the article in any way. The original article has been updated.

## Publisher's Note

All claims expressed in this article are solely those of the authors and do not necessarily represent those of their affiliated organizations, or those of the publisher, the editors and the reviewers. Any product that may be evaluated in this article, or claim that may be made by its manufacturer, is not guaranteed or endorsed by the publisher.

