# Supplementary material for: Dynamics of Emergency Cardiovascular Hospital Admissions and In-Hospital Mortality During the COVID-19 Pandemic: Time Series Analysis and Impact of Socioeconomic Factors
Source: Front Cardiovasc Med. 2022 Apr 26;9:827212. doi: 10.3389/fcvm.2022.827212 (PMC9087754; doi:10.3389/fcvm.2022.827212)
Supplement: Supplementary file 1 [file Data_Sheet_1.docx]

Table of contents

[Supplementary Table 1. ICD-10 Diagnostic and Procedure Codes 2](#_Toc85447193)

[Supplementary table 2. Characteristics of patients admitted to hospital for ACS and acute HF in the preCOVID-19 and the COVID-19 periods 3](#_Toc85447194)

[Supplementary Table 3. Negative binomial model parameters for the estimation of mean IRR of ACS admissions for each COVID-19 period as compared with the corresponding preCOVID-19 period. 4](#_Toc85447195)

[Supplementary Table 4. Negative binomial model parameters for the estimation of mean IRR of HF admissions for each COVID-19 period as compared with the corresponding preCOVID-19 period. 4](#_Toc85447196)

[Supplementary Table 5. Negative binomial model parameters for the estimation of weekly IRR of ACS admissions at each slope (fractional polynomic coefficients). 5](#_Toc85447197)

[Supplementary Table 6. Negative binomial model parameters for the estimation of weekly IRR of HF admissions at each slope (fractional polynomic coefficients). 5](#_Toc85447198)

[Supplementary figure 1. Observed minus expected number of hospitalizations (model residuals) for A) ACS, and B) HF, between 1 January 2019 and 31 December 2020. 6](#_Toc85447199)

[Supplementary figure 2. Model with fractional polynomic coefficients for ACS 8](#_Toc85447200)

[Supplementary figure 3. Model with fractional polynomic coefficients for HF 8](#_Toc85447201)

[Supplementary figure 4. Weekly Acute Coronary Syndrome episodes in women 9](#_Toc85447202)

[Supplementary figure 5. Weekly Acute Coronary Syndrome episodes in men 9](#_Toc85447203)

[Supplementary figure 6. Weekly Acute Coronary Syndrome episodes in ≥80 years’ old 10](#_Toc85447204)

[Supplementary figure 7. Weekly Acute Coronary Syndrome episodes in <80 years’ old 10](#_Toc85447205)

[Supplementary figure 8. Weekly Acute Coronary Syndrome episodes in the low income tercile 11](#_Toc85447206)

[Supplementary figure 9. Weekly Acute Coronary Syndrome episodes in the high income tercile 11](#_Toc85447207)

[Supplementary figure 10. Weekly Acute Heart Failure episodes in women 12](#_Toc85447208)

[Supplementary figure 11. Weekly Acute Heart Failure episodes in men 12](#_Toc85447209)

[Supplementary figure 12. Weekly Acute Heart Failure episodes in ≥80 years’ old 13](#_Toc85447210)

[Supplementary figure 13. Weekly Acute Heart Failure episodes in <80 years’ old 13](#_Toc85447211)

[Supplementary figure 14. Weekly Acute Heart Failure episodes in the low income tercile 13](#_Toc85447212)

[Supplementary figure 15. Weekly Acute Heart Failure episodes in the high income tercile 14](#_Toc85447213)

# Supplementary Table 1. ICD-10 Diagnostic and Procedure Codes

| Acute Coronary Syndrome |  |
| --- | --- |
| ST elevation | I210, I211, I212, I213 |
| Non ST elevation | I214, I222 |
| Other acute myocardial infarction | I219, I220, I221, I228, I229 |
| Unstable angina | I200 |
| Other Acute coronary syndrome | I248, I249 |
| Congestive heart failure | I501, I5021, I5022, I5023, I5030, I5031, I5032, I5033, I5040, I5041, I5042, I5043, I509, I110, I130, I97130, I97131, P290 |
| Percutaneous Coronary Angioplasty | 0270346, 027034Z, 0270356, 027035Z, 0270366, 027036Z, 0270376, 027037Z, 02703D6, 02703DZ, 02703E6, 02703EZ, 02703F6, 02703FZ, 02703G6, 02703GZ, 02703T6, 02703TZ, 02703Z6, 02703ZZ, 0271346, 027134Z, 0271356, 027135Z, 0271366, 027136Z, 0271376, 027137Z, 02713D6, 02713DZ, 02713E6, 02713EZ, 02713F6, 02713FZ, 02713G6, 02713GZ, 02713T6, 02713TZ, 02713Z6, 02713ZZ, 0272346, 027234Z, 0272356, 027235Z, 0272366, 027236Z, 0272376, 027237Z, 02723D6, 02723DZ, 02723E6, 02723EZ, 02723F6, 02723FZ, 02723G6, 02723GZ, 02723T6, 02723TZ, 02723Z6, 02723ZZ, 0273346, 027334Z, 0273356, 027335Z, 0273366, 027336Z, 0273376, 027337Z, 02733D6, 02733DZ, 02733E6, 02733EZ, 02733F6, 02733FZ, 02733G6, 02733GZ, 02733T6, 02733TZ, 02733Z6, 02733ZZ |

# Supplementary table 2. Characteristics of patients admitted to hospital for ACS and acute HF in the preCOVID-19 and the COVID-19 periods

|  | **ACS**  **N=8636** | | | **HF**  **N=27,566** | | |
| --- | --- | --- | --- | --- | --- | --- |
|  | PreCOVID-19 | COVID-19 | P value | PreCOVID-19 | COVID-19 | P value |
| N | 5503 | 3133 |  | 18845 | 8721 |  |
| Women | 1837 (33.38) | 1027 (32.78) | 0.568 | 10404 (55.21) | 4735 (54.29) | 0.156 |
| Age ≥80 | 1668 (30.31) | 837 (26.72) | <0.001 | 11655 (61.85) | 5203 (59.66) | 0.001 |
| Type of ACS |  |  | <0.001 |  |  |  |
| Unstable angina | 840 (15.26) | 531 (16.95) |  | - | - |  |
| NSTEMI | 3828 (69.56) | 2023 (64.57) |  | - | - |  |
| STEMI | 672 (12.21) | 217 (6.93) |  | - | - |  |
| Other MI | 89 (1.62) | 284 (9.06) |  | - | - |  |
| Other ACS | 74 (1.34) | 78 (2.49) |  | - | - |  |
| AMG weight, mean (SD) | 30.80 (17.22) | 18.45 (14.60) | <0.001 | 46.16 (16.60) | 30.74 (17.29) | <0.001 |
| Concomitant or previous coronavirus infection | - |  |  | - | 216 (2.48) |  |
| PCSA index, mean (SD) | 42.17 (14.54) | 41.79 (14.87) | 0.245 | 40.93 (15.16) | 41.50 (15.00) | 0.004 |
| Quantiles of PCSA index |  |  | 0.869 |  |  | 0.111 |
| 1st | 1298 (23.59) | 734 (23.43) |  | 5047 (26.78) | 2213 (25.38) |  |
| 2nd | 1309 (23.79) | 765 (24.42) |  | 4572 (24.26) | 2105 (24.12) |  |
| 3rd | 1319 (23.97) | 761 (24.29) |  | 4053 (21.51) | 1944 (22.29) |  |
| 4th | 1444 (26.24) | 793 (25.31) |  | 4611 (24.47) | 2198 (25.20) |  |
| PCI during hospitalization | 2492 (45.28) | 1320 (42.13) | 0.005 | 162 (0.86) | 72 (0.83) | 0.774 |
| Inhospital mortality | 281 (5.11) | 168 (5.36) | 0.606 | 1463 (4.76) | 598 (6.86) | 0.008 |
| Hospital length of stay (days), mean (SD); median (p25 - p75) | 9.32 (8.76);  7 (4-11) | 8.03 (7.05);  6 (4-10) | <0.001 | 9.86 (9.21);  8 (4-12) | 8.74 (7.51);  7 (4-11) | <0.001 |

**Supplementary Table 3. Negative binomial model parameters for the estimation of mean IRR of ACS admissions for each COVID-19 period as compared with the corresponding preCOVID-19 period.**

|  | **Coefficient** | **Std. Error** | **p-value** | **IRR** | **Lower CI** | **Upper CI** |
| --- | --- | --- | --- | --- | --- | --- |
| (Intercept) | 4.30 | 0.09 | <0.001 |  |  |  |
| Autoregressive term | 0.00 | 0.00 | 0.017 |  |  |  |
| Sin (2πt/52.14) | 0.063 | 0.024 | 0.009 |  |  |  |
| Cos (2πt/52.14) | 0.072 | 0.023 | 0.001 |  |  |  |
| Sin (4πt/52.14) | -0.069 | 0.023 | 0.003 |  |  |  |
| Cos (4πt/52.14) | 0.008 | 0.023 | 0.727 |  |  |  |
| First wave period | -0.41 | 0.07 | <0.001 | 0.66 | 0.58 | 0.76 |
| Between waves period | -0.08 | 0.04 | 0.057 | 0.92 | 0.84 | 1.00 |
| Second wave period | -0.23 | 0.05 | <0.001 | 0.80 | 0.72 | 0.88 |

# Supplementary Table 4. Negative binomial model parameters for the estimation of mean IRR of HF admissions for each COVID-19 period as compared with the corresponding preCOVID-19 period.

|  | | **Coefficient** | | **Std. Error** | | **p-value** | **IRR** | **Lower CI** | | **Upper CI** | |  |
| --- | --- | --- | --- | --- | --- | --- | --- | --- | --- | --- | --- | --- |
| (Intercept) | | 5.35 | | 0.08 | | <0.001 |  |  | |  | |  |
| Autoregressive term | 0.00 | | 0.00 | | <0.001 | |  | |  | |  | |
| Sin (2πt/52.14) | 0.142 | | 0.022 | | <0.001 | |  | |  | |  | |
| Cos (2πt/52.14) | 0.122 | | 0.018 | | <0.001 | |  | |  | |  | |
| First wave period | | -0.49 | | 0.06 | | <0.001 | 0.61 | 0.55 | | 0.69 | |  |
| Between waves period | | -0.21 | | 0.05 | | <0.001 | 0.81 | 0.74 | | 0.89 | |  |
| Second wave period | | -0.27 | | 0.05 | | <0.001 | 0.76 | 0.69 | | 0.84 | |  |

# Supplementary Table 5. Negative binomial model parameters for the estimation of weekly IRR of ACS admissions at each slope (fractional polynomic coefficients).

|  | **Coefficient** | | | | **Std. Error** | **p-value** | | | **IRR** | | **Lower CI** | | **Upper CI** | |
| --- | --- | --- | --- | --- | --- | --- | --- | --- | --- | --- | --- | --- | --- | --- |
| (Intercept) | | 4.49 | | | 0.07 | <0.001 | | |  | |  | |  | |
| Autoregressive term | | 0.00 | | | 0.00 | 0.690 | | |  | |  | |  | |
| Sin (2πt/52.14) | | | 0.063 | 0.024 | | | 0.009 |  | |  | |  | |  |
| Cos (2πt/52.14) | | | 0.072 | 0.023 | | | 0.001 |  | |  | |  | |  |
| Sin (4πt/52.14) | | | -0.069 | 0.023 | | | 0.003 |  | |  | |  | |  |
| Cos (4πt/52.14) | | | 0.008 | 0.023 | | | 0.727 |  | |  | |  | |  |
| From week 61 to week 64 | | -0.21 | | | 0.02 | <0.001 | | | 0.81 | | 0.78 | | 0.84 | |
| From week 65 to week 78 | | 0.28 | | | 0.03 | <0.001 | | | 1.32 | | 1.25 | | 1.39 | |
| From week 79 to week 91 | | -0.08 | | | 0.01 | <0.001 | | | 0.92 | | 0.90 | | 0.94 | |
| From week 92 to end | | -0.01 | | | 0.01 | 0.590 | | | 0.99 | | 0.97 | | 1.02 | |

# Supplementary Table 6. Negative binomial model parameters for the estimation of weekly IRR of HF admissions at each slope (fractional polynomic coefficients).

|  | | **Coefficient** | **Std. Error** | | | **p-value** | **IRR** | | | **Lower CI** | **Upper CI** |
| --- | --- | --- | --- | --- | --- | --- | --- | --- | --- | --- | --- |
| (Intercept) | | 5.66 | 0.06 | | | <0.001 |  | | |  |  |
| Autoregressive term | | 0.00 | 0.00 | | | 0.350 |  | | |  |  |
| Sin (2πt/52.14) | 0.142 | | | 0.022 | <0.001 | | |  |  |  | |
| Cos (2πt/52.14) | 0.122 | | | 0.018 | <0.001 | | |  |  |  | |
| From week 61 to week 64 | | -0.39 | 0.03 | | | <0.001 | 0.68 | | | 0.64 | 0.71 |
| From week 65 to week 78 | | 0.54 | 0.04 | | | <0.001 | 1.72 | | | 1.60 | 1.85 |
| From week 79 to week 91 | | -0.20 | 0.02 | | | <0.001 | 0.82 | | | 0.79 | 0.85 |
| From week 92 to end | | 0.06 | 0.01 | | | <0.001 | 1.06 | | | 1.04 | 1.08 |

# Supplementary figure 1. Observed minus expected number of hospitalizations (model residuals) for A) ACS, and B) HF, between 1 January 2019 and 31 December 2020.


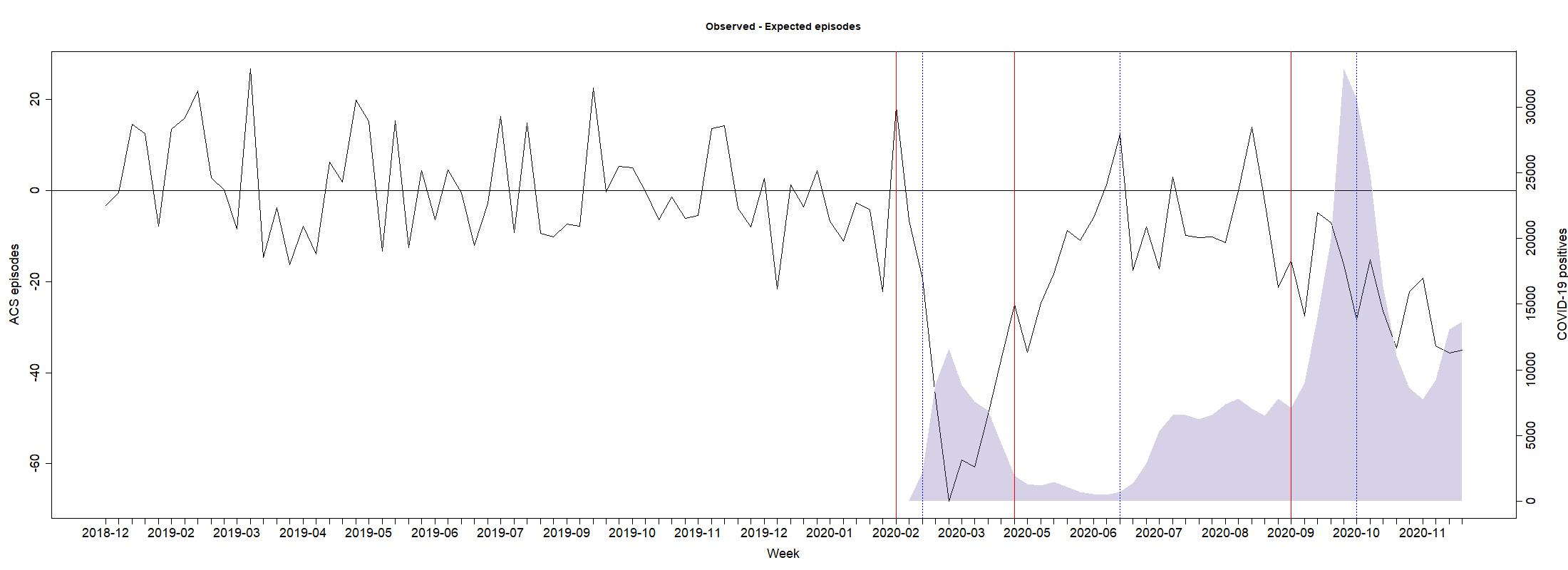


First lockdown (14/03/2020)

Gradual reopening (21/06/2020)

First COVID case in Catalonia (24/02/2020)

End of the first wave (27/04/2020)

Beginning of the second wave (21/09/2020)

Second lockdown (25/10/2020)

A)


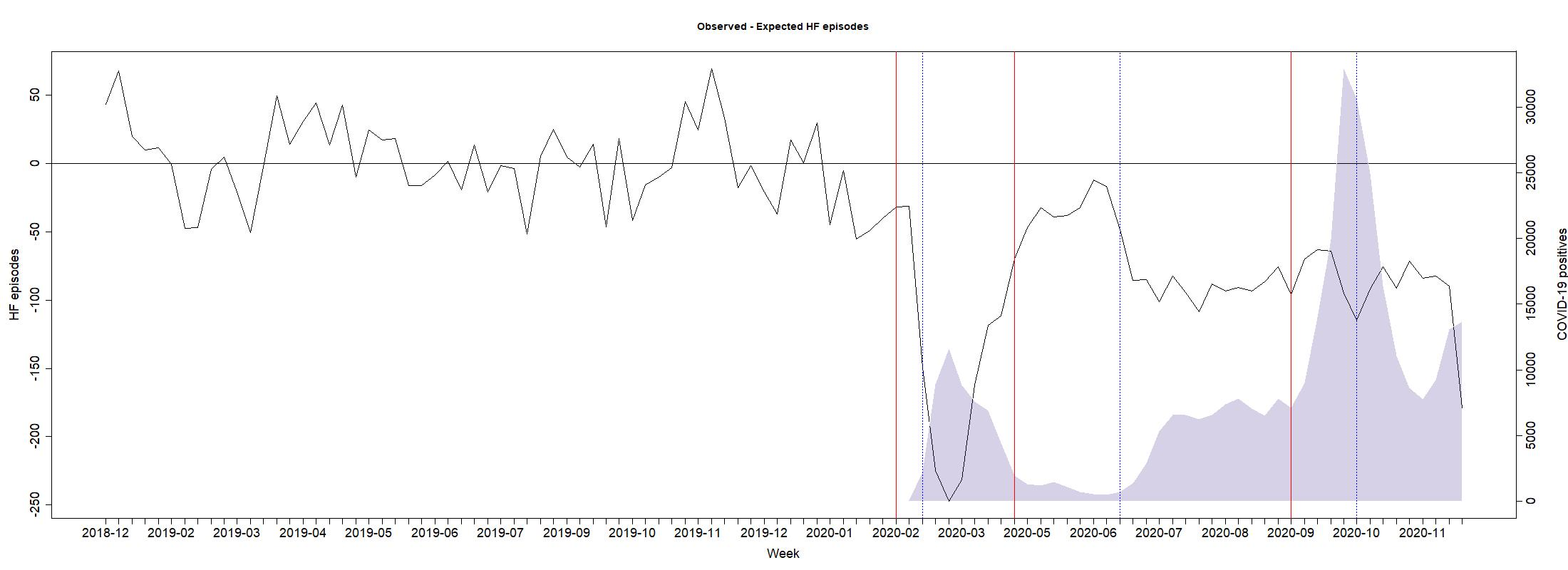


First lockdown (14/03/2020)

Gradual reopening (21/06/2020)

First COVID case in Catalonia (24/02/2020)

End of the first wave (27/04/2020)

Beginning of the second wave (21/09/2020)

Second lockdown (25/10/2020)

B)

# Supplementary figure 2. Model with fractional polynomic coefficients for ACS


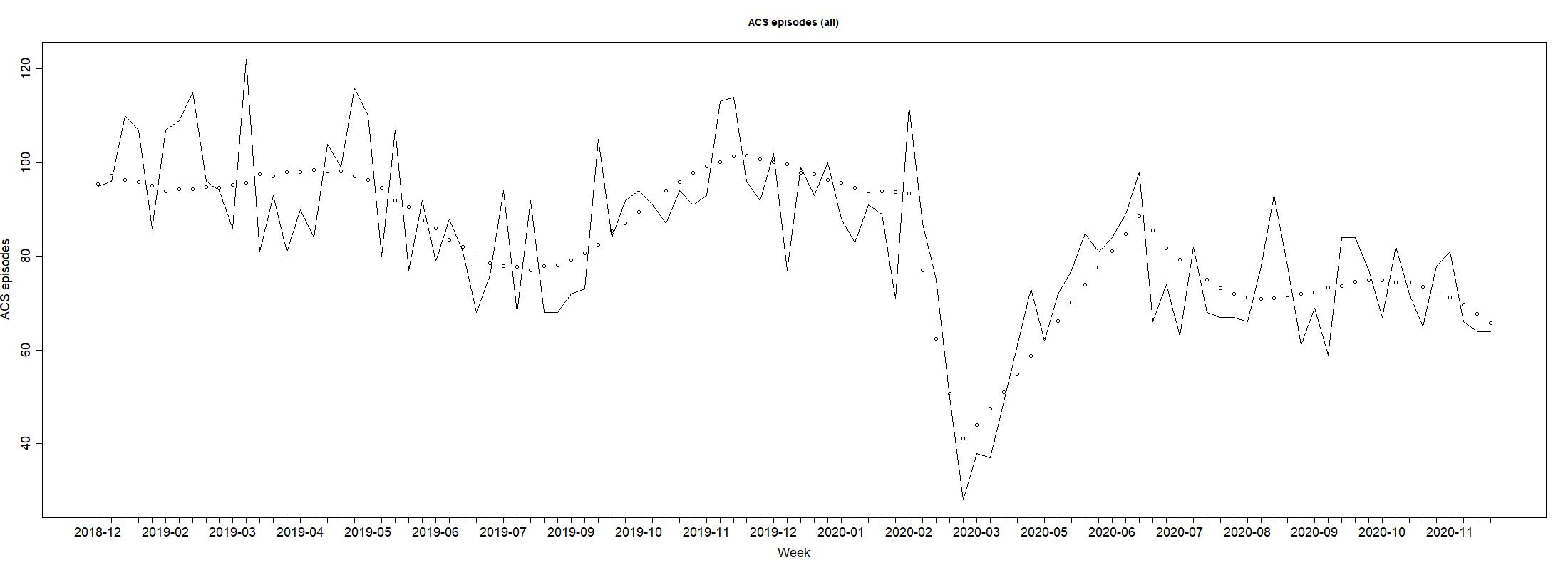


# Supplementary figure 3. Model with fractional polynomic coefficients for HF


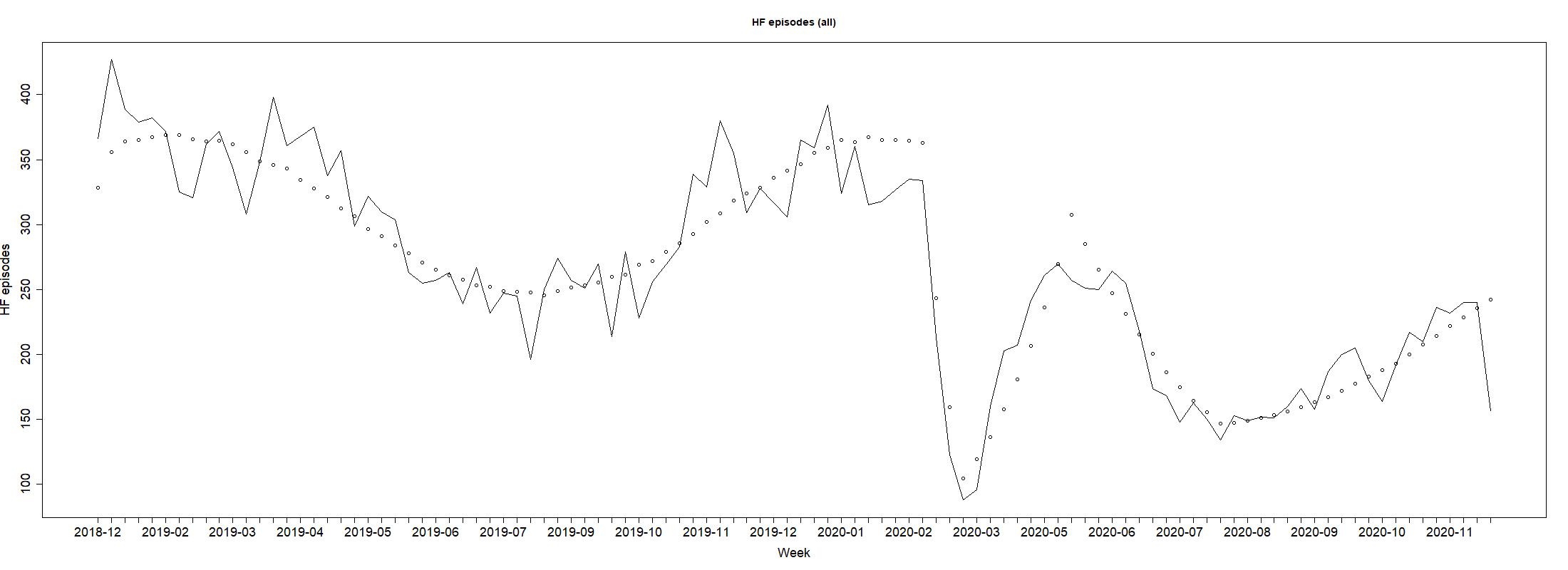


# Supplementary figure 4. Weekly Acute Coronary Syndrome episodes in women


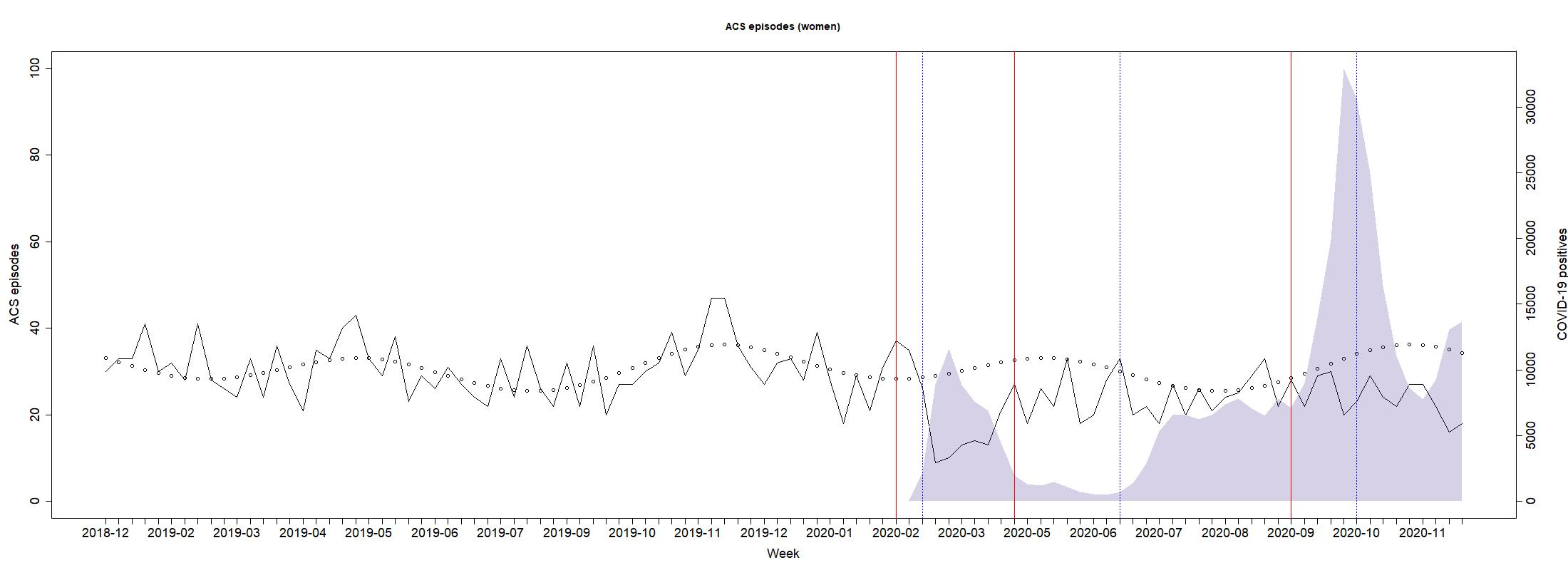


# Supplementary figure 5. Weekly Acute Coronary Syndrome episodes in men
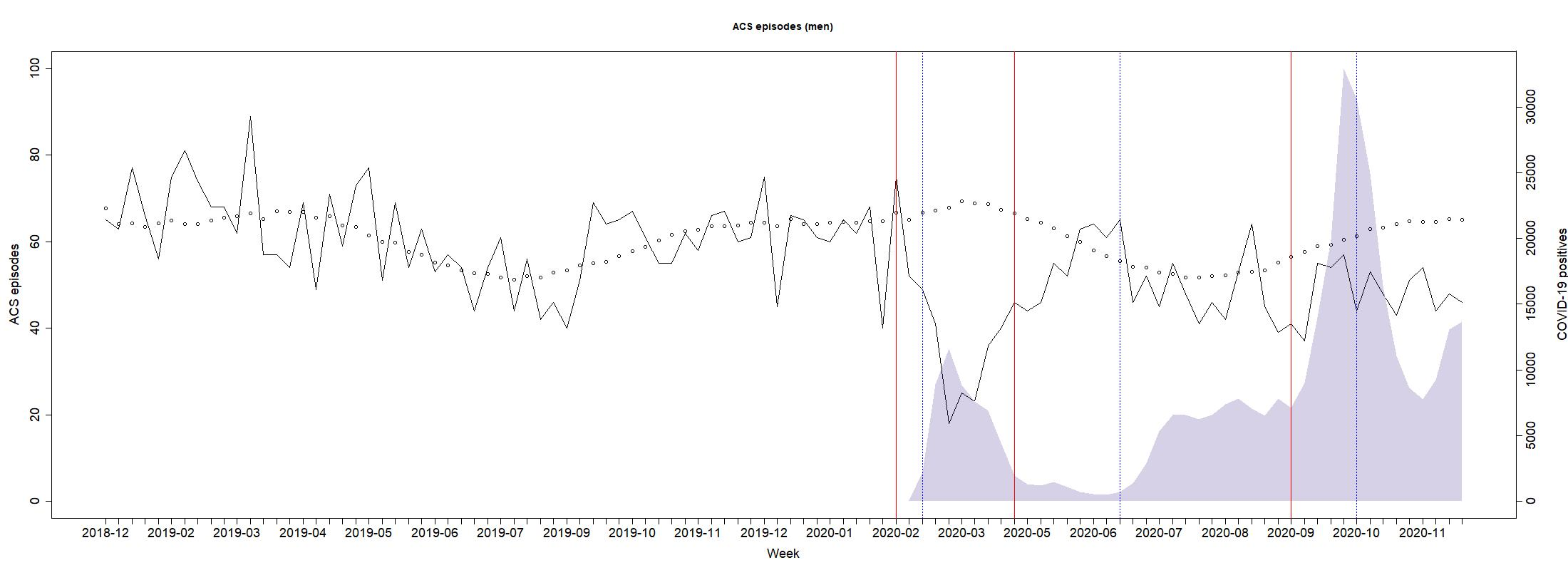


# Supplementary figure 6. Weekly Acute Coronary Syndrome episodes in ≥80 years’ old


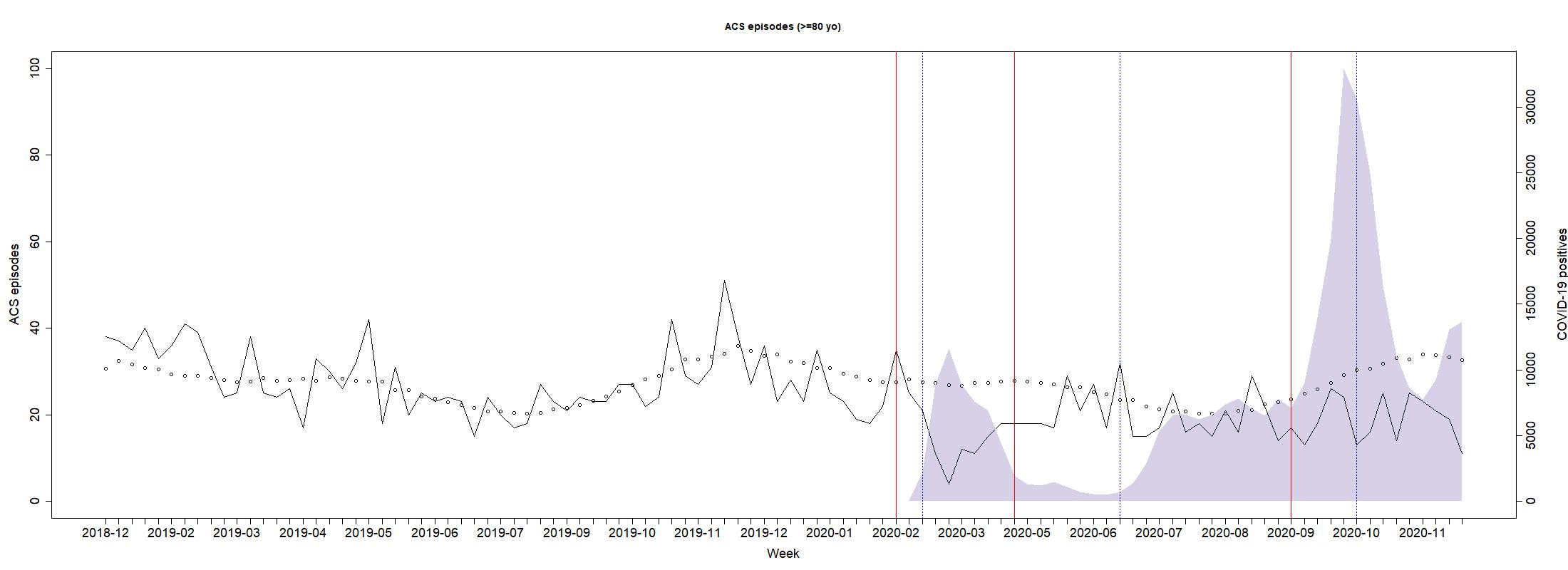


#

# Supplementary figure 7. Weekly Acute Coronary Syndrome episodes in <80 years’ old


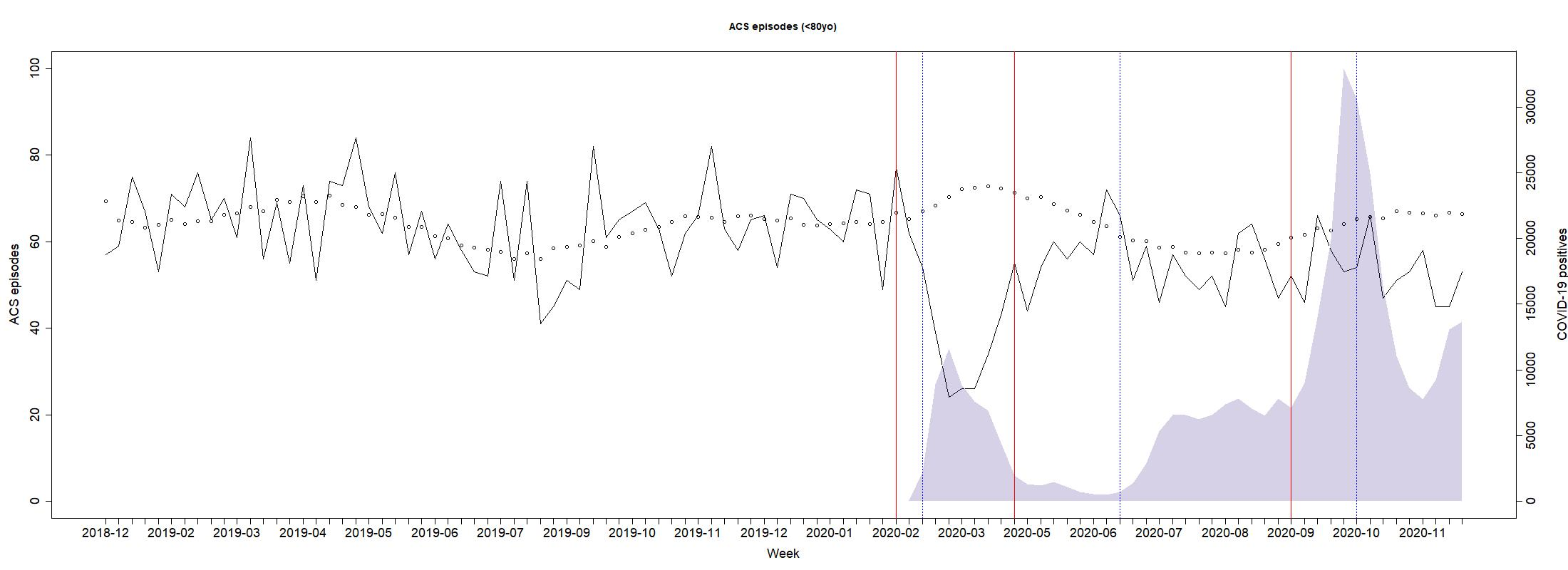


#

# Supplementary figure 8. Weekly Acute Coronary Syndrome episodes in the low income tercile


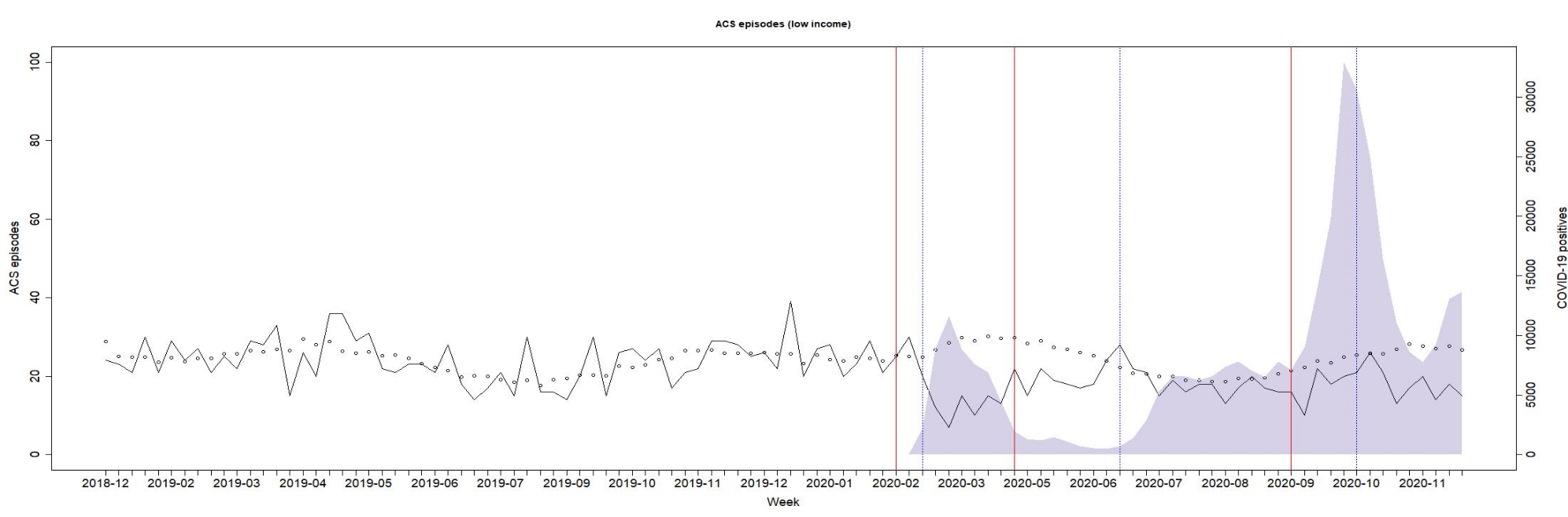


#

# Supplementary figure 9. Weekly Acute Coronary Syndrome episodes in the high income tercile


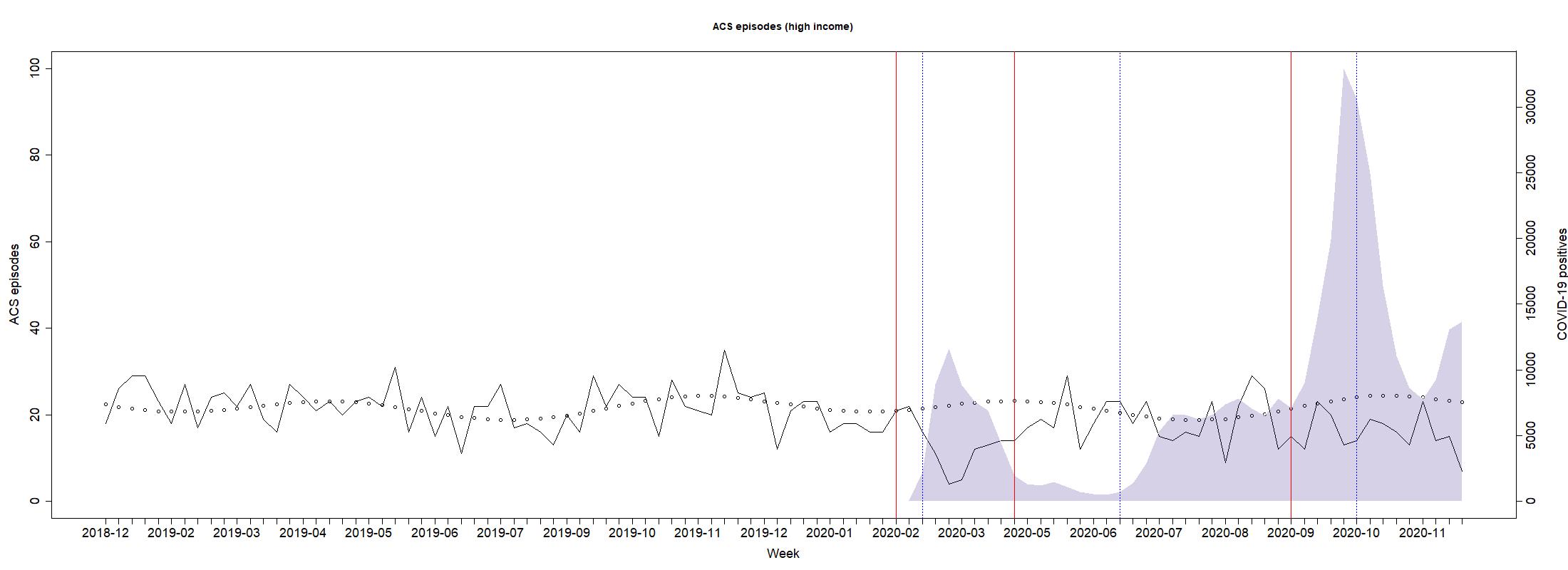


# Supplementary figure 10. Weekly Acute Heart Failure episodes in women


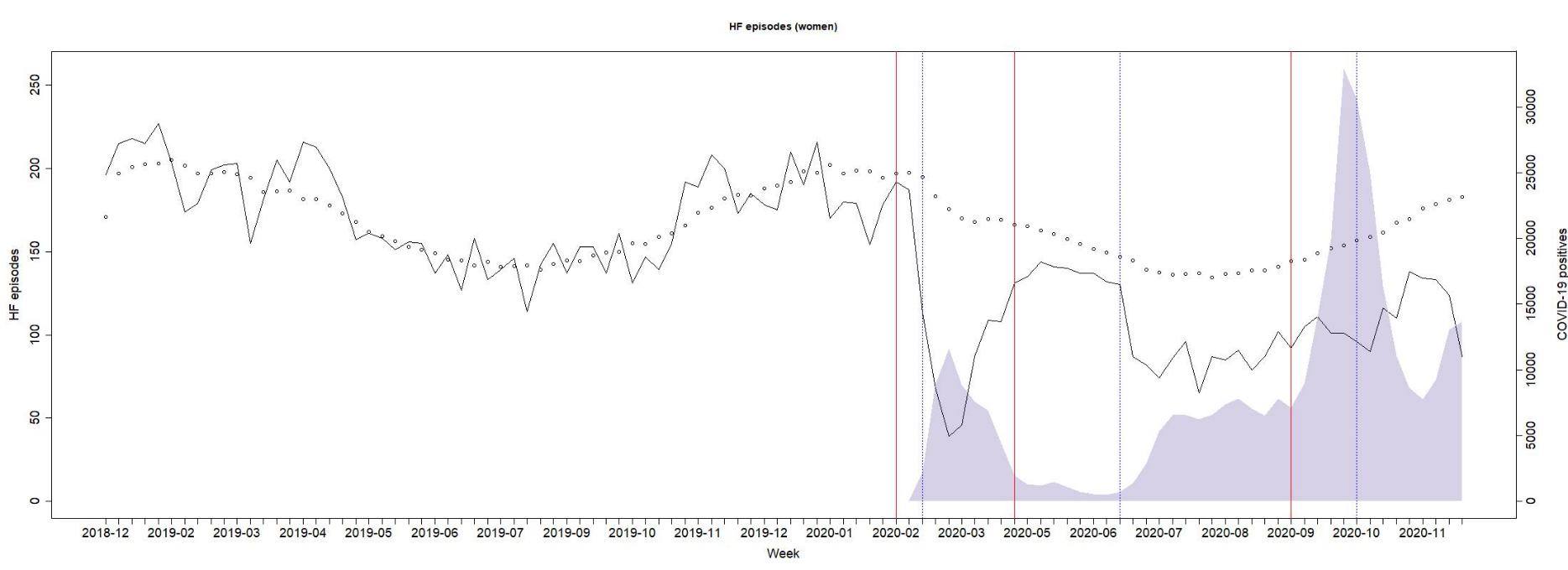


# Supplementary figure 11. Weekly Acute Heart Failure episodes in men


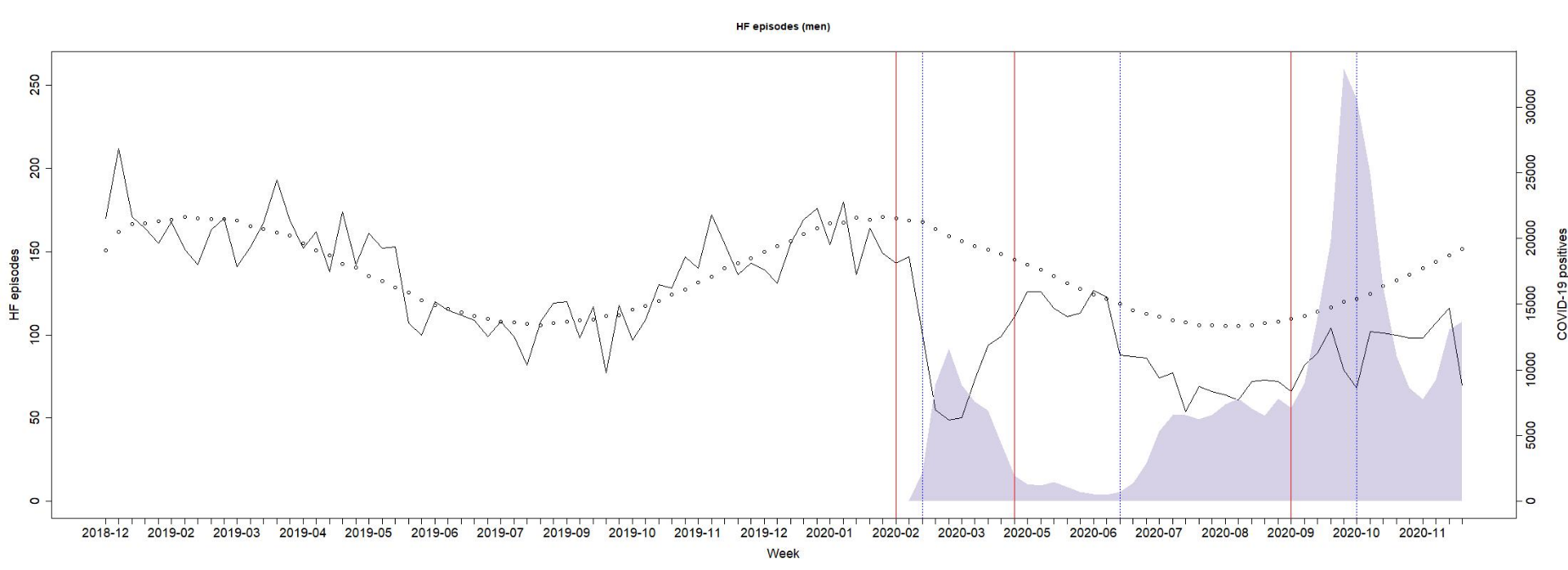


# Supplementary figure 12. Weekly Acute Heart Failure episodes in ≥80 years’ old


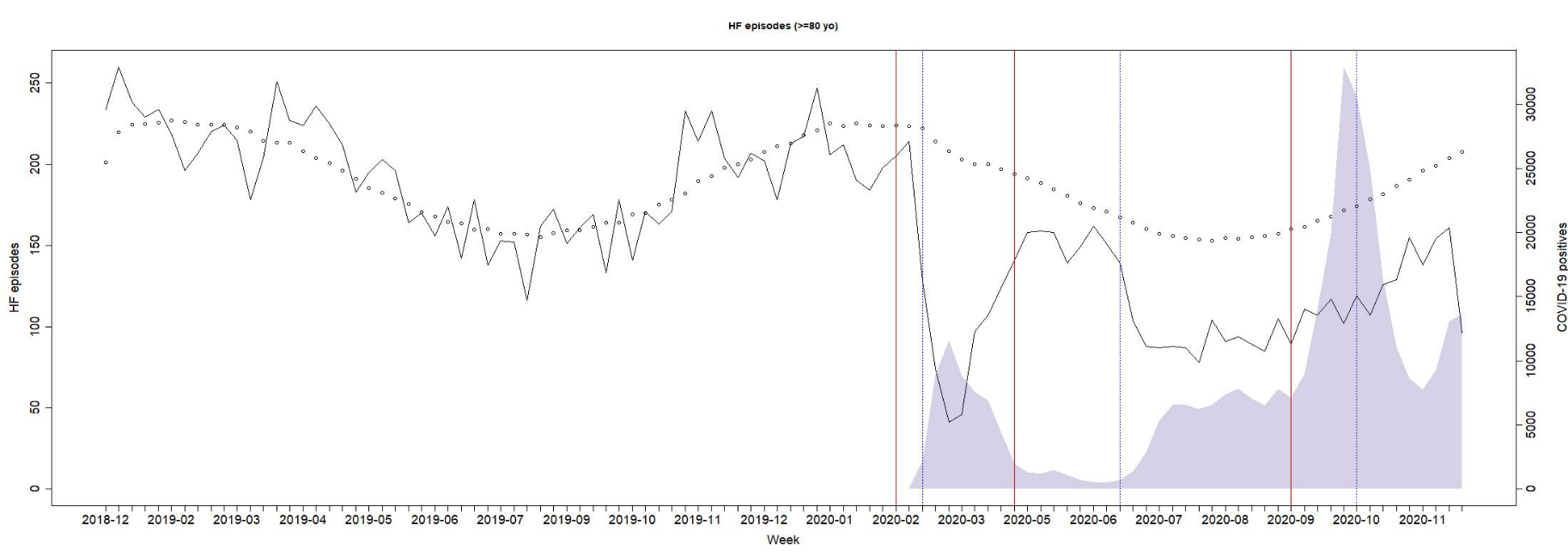


# Supplementary figure 13. Weekly Acute Heart Failure episodes in <80 years’ old

#
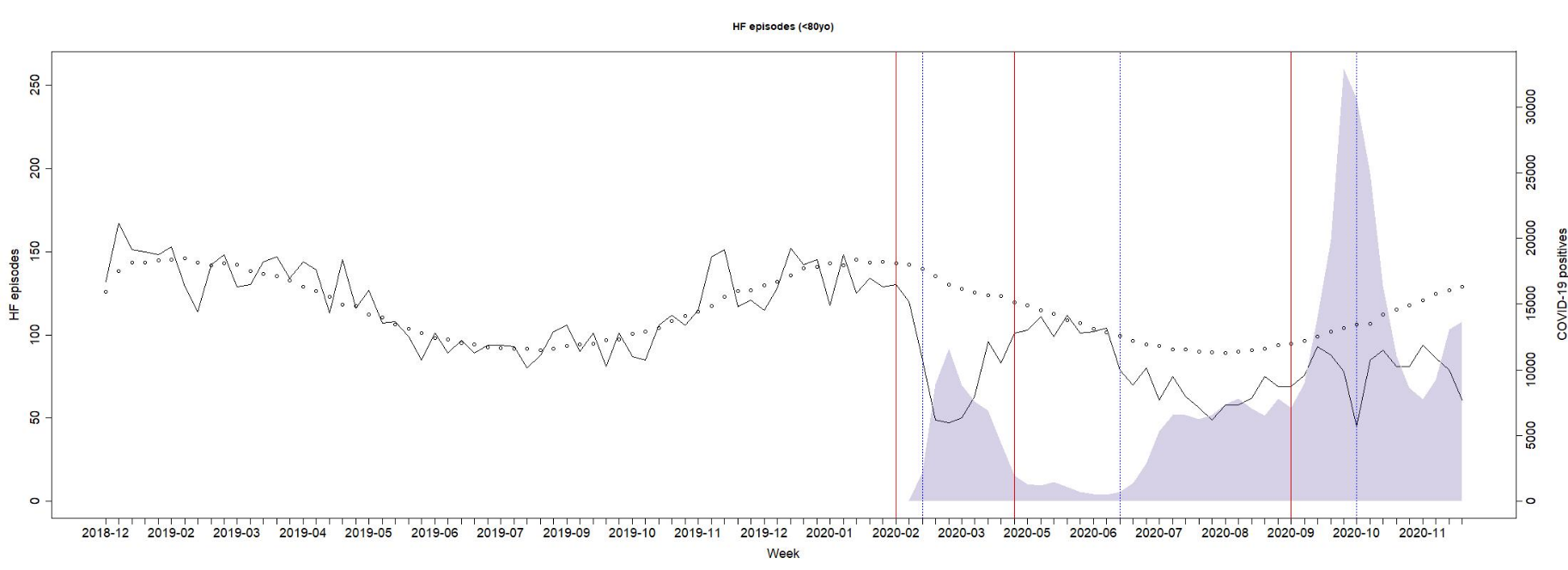
Supplementary figure 14. Weekly Acute Heart Failure episodes in the low income tercile


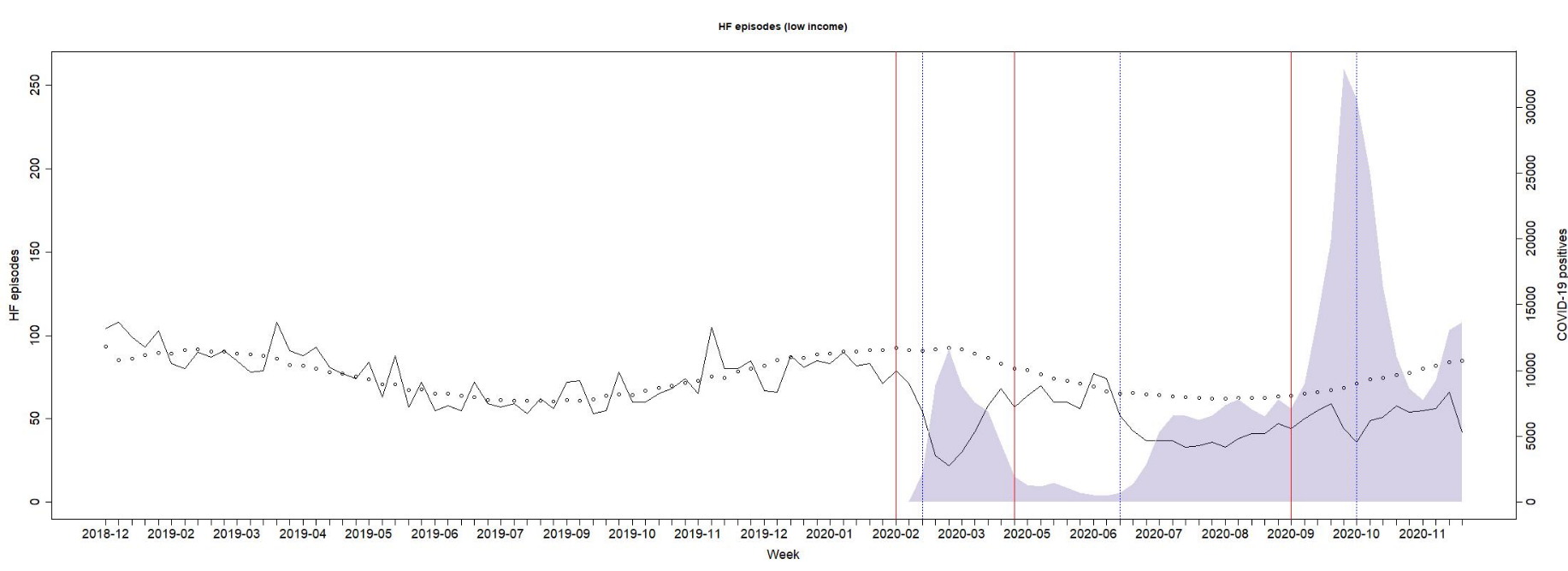


#

#
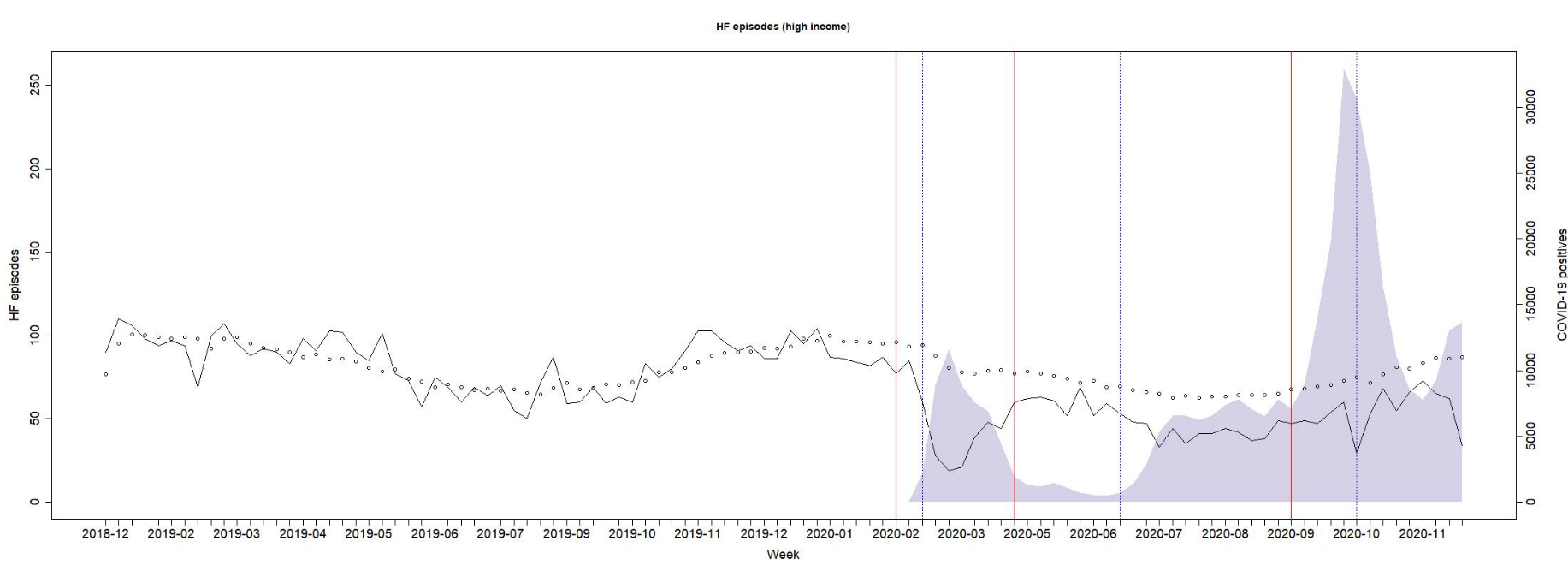
Supplementary figure 15. Weekly Acute Heart Failure episodes in the high income tercile
